# Neural and motor mechanisms of handwriting: from healthy aging to neurodegenerative disorders

**DOI:** 10.3389/fnagi.2026.1758541

**Published:** 2026-03-24

**Authors:** Francesca Burgio, Rachele Pezzetta, Jolien Gooijers, Sara Nordio, Dante Mantini

**Affiliations:** 1 Neuropsychology Laboratory, IRCCS San Camillo Hospital, Venice, Italy; 2 Integrated Research in Aging and Neurological Disorders Laboratory, IRCCS San Camillo Hospital, Venice, Italy; 3 Movement Control and Neuroplasticity Research Group, KU Leuven, Leuven, Belgium; 4 Leuven Brain Institute, KU Leuven, Leuven, Belgium; 5 Communication and Swallowing Laboratory, IRCCS San Camillo Hospital, Venice, Italy

**Keywords:** aging, brain network, cognitive decline, handwriting, motor control

## Abstract

Handwriting is a complex cognitive and motor skill supported by a distributed brain network involving cortical, subcortical, and cerebellar regions responsible for planning, execution, and sensorimotor integration. Beyond its communicative role, handwriting provides biologically meaningful information about brain function and motor control, serving as a sensitive marker of both normal and pathological changes. Age-related alterations, such as reduced fine motor precision, impaired sensory feedback, and cognitive slowing, contribute to the progressive decline in handwriting fluency and legibility. Importantly, distinctive handwriting patterns may be associated with early signs of neurodegenerative diseases, including Parkinson’s disease, Alzheimer’s disease, and Multiple Sclerosis, reflecting disease-specific alterations in motor and cognitive circuits. Advances in digital technology now enable high-resolution, quantitative analysis of handwriting kinematics, offering promising and scalable tools for diagnosis, longitudinal monitoring, and personalized rehabilitation. Furthermore, interventions incorporating fine motor and visuomotor coordination exercises, adaptive writing, and cognitive training may help preserve handwriting abilities and promote adaptive neural changes. In this review, we synthesize current evidence on the neural, behavioral, and technological mechanisms underlying handwriting across aging and neurodegenerative conditions. We provide an integrated overview of neural substrates, age- and disease-related alterations, and emerging digital approaches for assessment and intervention, highlighting their relevance for research and clinical practice. Overall, handwriting has the potential to offer a powerful, non-invasive window into brain health, bridging neuroscience, aging research, and digital medicine.

## 1 Introduction

Handwriting is a fundamental human skill that intertwines cognitive, sensory, and motor processes to produce legible and meaningful text (Tseng and Cermak, 1993; Lee et al., 2022). Unlike simple motor tasks, handwriting is a complex activity that requires the integration of visual, proprioceptive, and tactile feedback with precise motor control functions. It involves a widespread network of brain regions, each contributing to the planning, execution, and fine-tuning of hand movements (Planton et al., 2013; Baumann et al., 2022).

As individuals age, various physiological and cognitive changes can adversely affect their handwriting abilities (Asci et al., 2022). These changes are driven by structural and functional neural alterations, including volumetric reductions, cortical thinning, microstructural degradation of white matter tracts, altered neurotransmitter levels, as well as peripheral changes such as declines in muscle strength and sensory processing (Zia et al., 2021; Jin and Cai, 2023; Zhang et al., 2023). As a result, the fine motor control required for smooth and consistent handwriting progressively diminishes (Krampe, 2002), leading to alterations in speed, pressure, and legibility.

Importantly, similar mechanisms are also central to the development of neurodegenerative disorders, in which handwriting alterations often represent early and sensitive clinical signs. Conditions such as Parkinson’s disease (PD), Alzheimer’s disease (AD), and Multiple Sclerosis (MS) are characterized by progressive disruption of motor, cognitive, and sensorimotor integration processes that are essential for writing. These impairments can produce disease-specific handwriting patterns, including micrographia and tremulous strokes in PD (Heremans et al., 2016; Thomas et al., 2017), spatial disorganization and irregular letter formation in AD (Silveri et al., 2007; Fernandes et al., 2023), and reduced fluency or variable pen pressure in MS (Bisio et al., 2017).

From this perspective, aging and neurodegeneration can be viewed along a continuum of neural and behavioral change, in which normal age-related decline may interact with or exacerbate disease-related processes. Understanding the similarities and differences between these mechanisms is therefore essential for identifying early biomarkers, improving diagnostic sensitivity, and developing targeted rehabilitation strategies. Because handwriting integrates motor execution, sensory feedback, and higher-order cognition, it provides a noninvasive and potentially informative window into both physiological aging and pathological brain processes (Garre-Olmo et al., 2017; Thebaud et al., 2025).

In addition to traditional methods of handwriting assessment, such as visual inspection of legibility, pen-and-paper kinematic measurements, and standardized handwriting tests, there have been significant recent advances in digital technology. Digital platforms enable detailed analysis of handwriting dynamics, providing insights into fine motor functions that are not discernible through visual inspection alone (Guinet and Kandel, 2010; Chesnet et al., 2024; Vysakha et al., 2024). These tools facilitate remote monitoring and personalized intervention strategies, making them particularly valuable for assessing aging populations and detecting early signs of neurodegenerative conditions. Early and objective monitoring through digital tools enables the timely identification of subtle changes in motor and cognitive function.

Understanding how normal aging and neurodegenerative processes affect handwriting is useful in designing rehabilitation programs for older adults, while insights into disease-specific alterations can inform targeted interventions for individuals affected by neurological conditions, which may also occur at younger ages (Voelcker-Rehage, 2008). Such programs may include sensorimotor training, adaptive writing tools, and digital or computer-based activities designed to strengthen the neural substrates underlying handwriting and related cognitive functions. Despite growing interest in handwriting as both a neurocognitive marker and a potential target for therapeutic and training interventions, a comprehensive and integrated framework linking aging and neurodegeneration remains limited. Moreover, the considerable methodological diversity in handwriting assessment highlights the need for careful comparison across studies, an issue this review seeks to address. Recent developments also emphasize the importance of multidimensional and multimodal approaches, integrating kinematic, kinetic, physiological, and neural measures to improve sensitivity and diagnostic specificity (Impedovo and Pirlo, 2019; Benredjem et al., 2025).

To this end, the present narrative review aims to synthesize current evidence on the neural and behavioral mechanisms underlying handwriting across the adult lifespan and in neurodegenerative conditions. In preparing this review, we considered peer-reviewed studies involving adult participants and examining motor, cognitive, or neural aspects of handwriting. Relevant literature was identified through searches in major scientific databases (e.g., PubMed, Web of Science, Scopus) using keywords related to *handwriting*, *aging*, *motor control*, *sensorimotor integration*, and *neurodegenerative diseases*, and was supplemented by examining reference lists of key articles. Specifically, this review: (i) examines how aging and disease alter the neural circuitry and motor processes supporting handwriting; (ii) explores current methodologies, including digital tools, that use handwriting for diagnostic and monitoring purposes; and (iii) discusses strategies and interventions aimed at maintaining or restoring handwriting efficiency through motor, cognitive, and technological approaches.

## 2 Neural mechanisms of handwriting

Handwriting engages a highly distributed and interactive neural system that integrates motor, sensory, and cognitive processes to generate precise and adaptable movements. Rather than functioning as independent modules, brain regions involved in handwriting operate as a dynamic network in which information is continuously exchanged through functional connectivity and feedback mechanisms. In the following sections, we first describe the main brain regions contributing to handwriting and then discuss the pathways and mechanisms through which these regions interact to support writing behavior.

Neuroimaging studies, particularly those using functional magnetic resonance imaging (fMRI), have demonstrated coordinated and temporally structured activity across cortical motor areas, parietal regions, basal ganglia, and the cerebellum during writing tasks (Horovitz et al., 2013; Karimpoor et al., 2018). These network interactions support the integration of motor planning, spatial guidance, sensory monitoring, and linguistic encoding, enabling flexible adaptation of handwriting to task demands and environmental constraints (Baumann et al., 2022).

Neural pathways link these regions with each other and to peripheral effectors, while continuous sensory feedback may enable real-time monitoring and correction of pen movements (Nackaerts et al., 2018). Cognitive and sensorimotor integration further ensures that linguistic plans are seamlessly translated into coordinated motor sequences (Horovitz et al., 2013). From this perspective, handwriting should be understood as a systems-level function emerging from the coordinated activity of distributed neural circuits rather than isolated regional contributions (Karimpoor et al., 2018). Examining how these components interact provides key insights into the mechanisms supporting handwriting performance and how they may be altered by aging and neurodegenerative conditions (Nackaerts et al., 2018).

### 2.1 Neural substrates of handwriting

**Figure 1:**
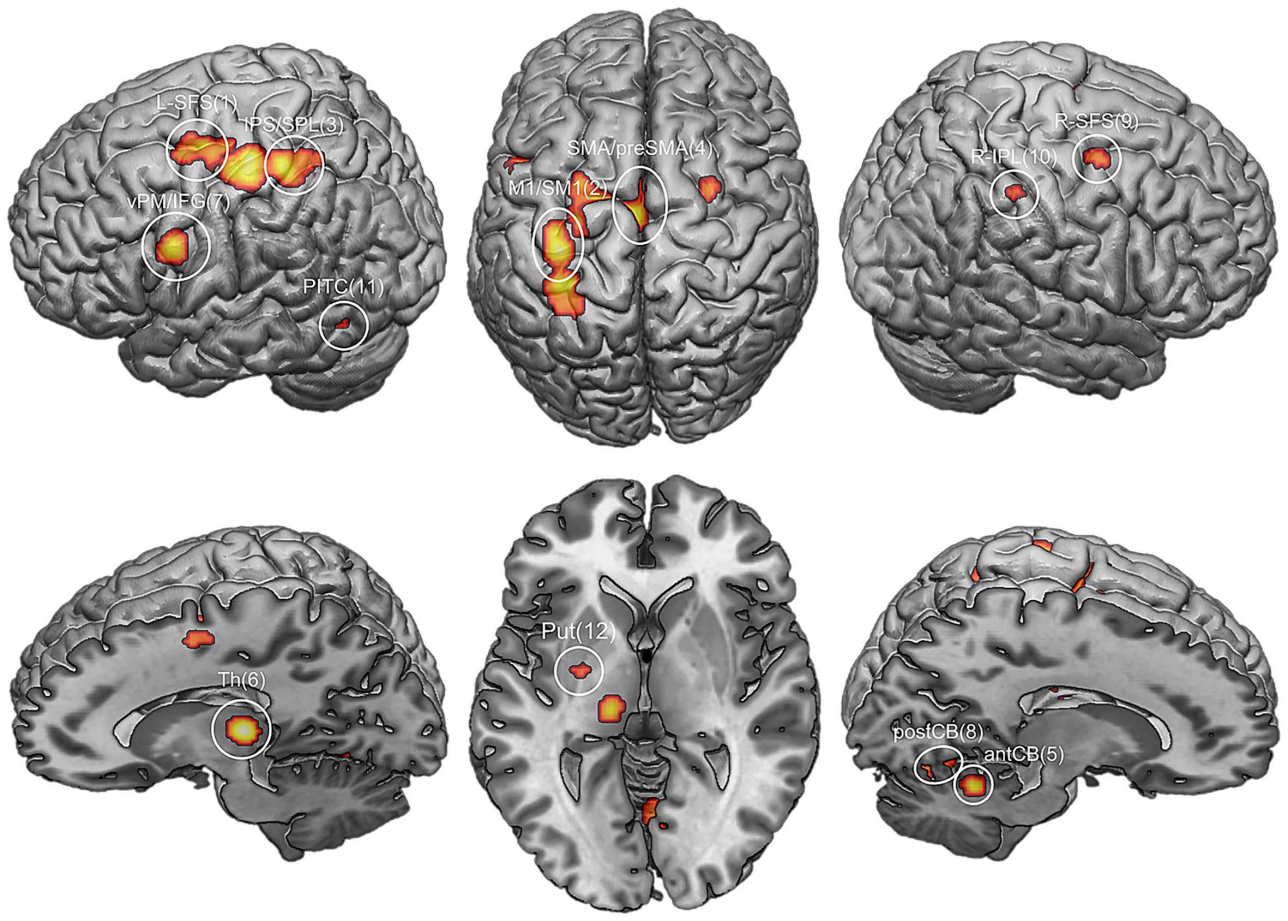
Results of a meta-analysis conducted with neuroimaging studies on handwriting. Brain regions involved in handwriting, numbered based on their relative reliability, are projected on a standard rendered template. 1. Left frontal superior sulcus (L-SFS); 2. Primary motor/sensorimotor cortex (M1/SM1); 3. Left inferior parietal lobule/superior parietal lobule (IPS/SPL); 4. Supplementary motor area (SMA)/pre-SMA; 5. Anterior cerebellum (antCB); 6. Thalamus (Th); 7. Left ventral premotor area/inferior frontal gyrus (vPM; IFG); 8. Posterior cerebellum (postCB); 9. Right superior frontal sulcus (R-SFS); 10. Right inferior parietal lobule (R-IPL); 11. Posterior inferior temporal cortex (PITC); 12. Putamen (Put). The figure is reproduced from Planton et al. (2013), with permission.

Handwriting relies on a distributed neural network in which motor, premotor, parietal, and subcortical regions interact dynamically to generate precise and adaptive movements. The production of fluid and legible written text requires the integration of cognitive planning, sensory feedback, and motor control within this network (Planton et al., 2013; Baumann et al., 2022). Key regions involved include the primary motor cortex, premotor cortex, supplementary motor area, basal ganglia, cerebellum, and parietal cortex (Figure 1). These regions are functionally connected and operate through coordinated activity patterns that support motor preparation, execution, error correction, and learning. The following sections describe the specific contributions of these components and how aging and neurological disorders may alter their function.

#### 2.1.1 Primary motor cortex (M1)

The primary motor cortex (M1), situated in the precentral gyrus, is extensively involved in voluntary movements, which are essential for handwriting. Writing is a fine motor task that requires the coordinated activation of specific muscles, driven by neural impulses originating from M1 (Scott, 2003; Chouinard and Paus, 2006). M1 is crucial for fine motor control and coordination of hand and finger movements, which are vital for writing legibly, including accurate letter or word formation. It also contributes to the smooth coordination of sequential motor patterns required for well-formed and legible writing (Scott, 2003; Horovitz et al., 2013; Planton et al., 2013).

As it plays a direct role in the handwriting process, M1 influences the legibility, smoothness, and consistency of written output, aspects that together determine handwriting quality and form. These features are highly individual and can vary across time and context, reflecting both personal motor habits and task demands. The functioning of M1 during aging may deteriorate due to multiple neurobiological changes, including synaptic loss, reduced cortical excitability, altered neurotransmitter balance, and declines in interhemispheric connectivity (Coppi et al., 2014; Bhandari et al., 2016). These alterations collectively contribute to reduced manual dexterity, among other motor impairments, emphasizing the need to understand how M1 degradation relates to age-associated changes in handwriting performance.

#### 2.1.2 Premotor cortex

Unlike M1, which controls the muscles required to perform a specific movement, the premotor cortex (PMC), positioned anterior to the M1, is important in organizing physical tasks (Chouinard and Paus, 2006; Gremel and Costa, 2013). This region coordinates the incoming sensory information to generate the motor programs necessary for writing. At the same time, the PMC selects a particular number and array of movements to compose rather well-coordinated and readable text (Longcamp et al., 2003). It encompasses the processes of joining letters and, more importantly, the motions required when transitioning from one letter to the next, which play a key role in the formation of neat handwriting.

The PMC further processes sensory feedback in collaboration with the primary motor cortex, cerebellum, and parietal regions to adjust ongoing movements and ensure that each pen stroke is accurately placed. Aging-related alterations in the PMC, such as reductions in gray matter volume, decreased functional connectivity with motor and parietal regions, and slower integration of sensory feedback, can impair the fine motor planning required for writing. These changes often manifest as clumsy, rough, or discontinuous pen movements, contributing to handwriting irregularities and reduced fluency (Rurak et al., 2021; Zapparoli et al., 2022).

#### 2.1.3 Supplementary motor area

The supplementary motor area (SMA) is defined as that part of the cerebral cortex lying at the medial part of the frontal lobe, and it plays a significant role in the initiation and regulation of movement in sequences (Tanji, 1994). The SMA plays an especially vital role in time-locked coordination of the movements involved in writing and in the bimanual coordinated movements that are needed, for example, during writing and holding a paper still (Wing, 2000).

Connection to other motor areas ensures that the movements needed for writing are well-timed and performed in a coordinated and efficient manner. In particular, the SMA contributes by coordinating the sensorimotor pathways that link cortical motor regions to spinal motor neurons, supporting the initiation and sequencing of writing movements. These pathways may gradually deteriorate with age, affecting both the initiation and execution of writing movements (Rurak et al., 2021). This can lead to hesitation, pauses, and asymmetry in prints or irregularities associated with handwriting rhythm (Pei et al., 2021).

Therefore, it is crucial to acknowledge the contribution of the SMA when developing strategies that help older adults practice and strengthen handwriting-related abilities, such as movement sequencing, timing, and bimanual coordination.

#### 2.1.4 Basal ganglia

The basal ganglia are a group of subcortical grey matter nuclei, which play a significant role in determining movement and force. These structures help regulate motor tasks such that people move appropriately (Dhawale et al., 2021). The basal ganglia assist in controlling muscular movements and regulating the repetitiveness and smoothness of handwriting by refining the motor plans supplied by the cortex (Phillips et al., 1993).

Movement is a complex process that involves, among other elements, dopaminergic neurons situated in the basal ganglia. Age-related declines in dopaminergic function can contribute to increased rigidity and tremulous movements, which share features with, but are distinct from, the motor symptoms observed in PD. These symptoms can substantially influence handwriting, as it may become smaller, more tremulous, and less legible (Thomas et al., 2017). Understanding the function of the basal ganglia in handwriting can aid in defining distinct motor anomalies characterizing the aging process and designing therapies that might slow down the detrimental effects.

#### 2.1.5 Cerebellum

The cerebellum, located in the posterior part of the brain, plays a key role in motor learning and, more specifically, in the regulation and fine-tuning of movements (Paulin, 1993). During handwriting, intrinsic muscle co-contractions not only facilitate movement but also help stabilize fine motor control, improving the timing of writing actions and allowing adjustments in response to sensory feedback. As the brain’s coordinator and adjuster involved in error correction, the cerebellum plays a crucial role in training for efficient and well-executed writing abilities (Horovitz et al., 2013). It enables fluent handwriting by continuously comparing predicted motor commands (efference copy) with incoming sensory feedback (afference) and adjusting motor output accordingly to minimize errors in movement timing and precision.

Age-related structural and functional decline in the cerebellum, including volume reduction, neuronal loss, and decreased connectivity, may manifest as impairments in coordination and movement sequencing; therefore, the writing may become less harmonized with more mistakes (Iskusnykh et al., 2024). This kind of problem manifests itself in terms of involuntary shaking of the arm, inconsistent pressure applied to the writing instrument, and problems in keeping letters and spaces of writing consistent in style (Lupo et al., 2019). Understanding the cerebellum’s role in handwriting provides insight into how age-related changes in this region affect motor function and lays the foundation for training activities aimed at maintaining or enhancing cerebellar involvement in older adults.

#### 2.1.6 Parietal cortex

There is strong evidence that the parietal cortex is involved in manual activities and handwriting, specifically the posterior parietal cortex. This region integrates and encodes proprioceptive and visual information to modify, control, and coordinate movements necessary to form letters and words on the page (Brownsett and Wise, 2010).

It is suggested that parietal lesions may lead to spatial disorientation (Verdon et al., 2010; Gillebert et al., 2011) and problems in spatial and sensory integration, resulting in poor alignment of handwriting. This can cause inconsistencies in the size of the letters on a page and the spaces between them, making the text less likely to be understood clearly (Menon and Desmond, 2001).

### 2.2 Neural pathways

Handwriting involves the coordinated activity of muscles and neural pathways that transmit both motor commands and sensory feedback from various brain regions. The corticospinal tract serves as a primary pathway linking the cerebral cortex to the spinal motor neurons, enabling precise and rapid muscle contractions necessary for writing (Cinelli et al., 2019).

In addition, thalamocortical and corticothalamic connections transfer sensory information to motor areas, allowing tactile and proprioceptive feedback from the hand and fingers to be accurately integrated and used to fine-tune ongoing movements. The cerebellum–thalamus–motor cortex circuit further refines these signals to produce smooth and coordinated handwriting (Aumann, 2002; Sherman, 2016).

Beyond these core circuits, several white matter tracts contribute to the integration of sensory, motor, and cognitive processes essential for handwriting. The Superior Longitudinal Fasciculus (SLF) connects frontal, parietal, and occipital regions, supporting visuomotor integration and spatial coordination (Thiebaut de Schotten et al., 2012; Budisavljevic et al., 2015). The Frontal Aslant Tract (FAT) links the inferior frontal gyrus with the supplementary motor area, facilitating movement initiation, sequencing, and fluency (Catani et al., 2012; Dick et al., 2014). The Cingulum participates in attention, executive control, and error monitoring processes that sustain adaptive handwriting performance (Jones et al., 2013; Bubb et al., 2018). Finally, the Corpus Callosum enables intermanual coordination and the transfer of motor information between the two hemispheres, supporting bimanual control during complex writing tasks (Wahl et al., 2007; Bonzano et al., 2008).

### 2.3 Sensory feedback mechanisms

Sensory feedback mechanisms play a crucial role in handwriting, which relies on precise and well-coordinated hand movements. In this context, sensory feedback mainly refers to proprioceptive and tactile information, including input about the position, movement, and pressure of the hand and fingers, which enables continuous adjustment of grip and trajectory during writing. While visual feedback also contributes to guiding letter formation, proprioceptive and touch-based feedback are particularly essential for maintaining accuracy, even when using unfamiliar writing instruments (Danna and Velay, 2015; Danna et al., 2022). Thus, sensory feedback helps the writer maintain control over hand and finger positioning, enabling the production of legible and accurate writing.

Hand movements are strongly coordinated by visual feedback, which ensures that letters and words are properly shaped, sized, and aligned on the page. When visual input is removed, handwriting performance deteriorates markedly, with increased spatial errors and irregular letter formation (van Galen, 1991; Teulings et al., 1997). Visual monitoring allows writers to track their progress and correct errors in real time, thereby maintaining overall clarity and legibility.

Reduced capabilities in processing sensory feedback can occur as a result of aging, and these changes can markedly interfere with the ability to sustain legible, fluent, and spatially accurate handwriting (Krampe, 2002; Zapparoli et al., 2022). Recognizing that sensory feedback is a key component of effective handwriting highlights the importance of preserving somatosensory function and providing targeted support for older adults.

### 2.4 Cognitive and sensorimotor integration

The sensorimotor aspects of handwriting involve components such as letter movement, hand and arm coordination and control, motor planning, and execution, which depend on the corticospinal and extrapyramidal tracts as well as various brain regions, particularly the parietal lobes. These regions are involved in the planning and execution of movement, and they play an important role in the integration of grammatical as well as motor planning abilities needed to transform thoughts into text (Cerni and Job, 2022, 2024).

Considering the cognitive dimension of handwriting, higher-order processes come into play to support planning, sequencing, and linguistic encoding. In this context, the prefrontal cortex is central to the executive skills required for writing, including organization, sequencing, and self-monitoring (Megumi et al., 2023), while Broca’s and Wernicke’s areas are crucial for language generation and comprehension (Blank et al., 2002).

Cognitive decline that may occur with healthy aging can affect the neural systems involved in handwriting, leading to difficulties in planning, organizing, and monitoring writing tasks. Such changes may alter the physical appearance of written text, for instance, through reduced legibility, irregular spacing, or fragmented sequencing, and can also impair the ability to produce coherent and well-structured written information (Ericsson et al., 1996; Asci et al., 2022). These effects reflect the interplay between cognitive and motor components of handwriting, where reduced executive control and working memory compromise motor planning and execution.

Understanding these mechanisms underscores the importance of designing interventions that simultaneously target cognitive and motor domains. In this context, rehabilitation should aim to reinforce functional connectivity within affected brain networks through restitutive, plasticity-based approaches (Kleim and Jones, 2008; Dayan and Cohen, 2011). This can be achieved by repetitive and meaningful practice tasks that promote co-activation of relevant neuronal circuits (“neurons that fire together, wire together”), thereby strengthening synaptic efficiency (Taubert et al., 2011). Complementary methods such as cognitive training and non-invasive brain stimulation (e.g., transcranial direct current stimulation or repetitive transcranial magnetic stimulation) may further enhance these effects, supporting both sensorimotor and executive aspects of handwriting in older adults (Polanía et al., 2011).

## 3 Effects of aging on handwriting

The aging process affects handwriting through a complex interplay of neural, sensory, and cognitive changes. As the brain and musculoskeletal system undergo structural and functional decline, the precision, fluidity, and legibility of handwriting gradually deteriorate. Age-related alterations in motor control, sensory feedback, and cognitive processing jointly contribute to slower, less coordinated writing movements and increased variability in stroke execution. Understanding these changes requires a multidimensional approach that examines their neural and muscular bases, the degradation of sensorimotor integration, and the impact of cognitive decline. The following sections discuss each of these domains in detail, outlining how they interact to shape handwriting performance in older adults.

### 3.1 Neural and muscular bases of handwriting decline in aging

With advancing age, handwriting performance and legibility decline progressively as a result of multiple interacting factors, including motor control impairments, sensory degradation, cognitive slowing, and muscular weakness (Krampe, 2002; Seidler et al., 2010). Age-related reductions in neuronal density and synaptic integrity occur across many cortical and subcortical regions, but changes within the motor cortex and basal ganglia are particularly relevant for handwriting because of their key roles in fine motor coordination and movement sequencing (Raz et al., 2005; Polanía et al., 2011). Degeneration in these areas leads to less precise and less efficient control of hand movements necessary for producing smooth, coordinated writing.

Another well-organized set of motor structures that degenerates with age is the basal ganglia. Dopamine tone in general declines with age, thus affecting the output of the basal ganglia on motor command optimization (Darbin, 2012). This decrease in dopaminergic output can lead to the slowness and shuffling movements of the hand, and in extreme cases, it is thought to contribute to diseases such as PD, in which micrographia, or writing with very small letters, and tremors may occur (Heremans et al., 2016; Thomas et al., 2017). In addition to these neurotransmitter-related changes, aging is also associated with reduced white-matter integrity in frontostriatal and sensorimotor pathways, which further disrupts the efficiency of motor signal transmission and contributes to declines in handwriting fluency and coordination (Gooijers et al., 2024).

Lack of muscle strength, or sarcopenia, also contributes to the deterioration of handwriting with age. With sarcopenia, writing can be problematic, particularly when it comes to creating a comfortable and stable grip, precise pressure on a writing instrument, and smooth-lined text (Sousa-Santos and Amaral, 2017). While the process of aging seems to have taken a toll on gross motor control, the rigidity and decrease in muscle tone can also be attributed to it.

### 3.2 Age-related changes in sensorimotor integration

Guided handwriting relies on a tight integration between sensory feedback from the limbs and the motor commands that generate writing movements. With aging, this communication becomes less efficient, partly due to structural and functional degeneration in the sensory–motor pathways, including reduced white matter integrity and slower neural transmission (Goble et al., 2009; Danna and Velay, 2015).

A key contributor to this process is proprioception, the body’s ability to sense the position and movement of its limbs, which provides continuous feedback necessary for fine motor adjustments during writing. Although proprioception is only one element of the broader sensorimotor integration system, its decline has a substantial impact on handwriting performance. With age, the sensitivity of proprioceptors in muscles and joints decreases, and information transfer through white matter tracts becomes less efficient, reducing the brain’s ability to precisely regulate hand and finger movements (Boisgontier and Swinnen, 2015). Consequently, movement accuracy and control decline, leading to reduced legibility, slower writing speed, and less fluid style in older adults.

Another important factor to consider is the decline in both the rate and quality of visual processing and the associated reduction in visuomotor coordination that occurs with aging. Beyond central neural changes, aging also affects the ocular system itself: near-focusing ability diminishes (presbyopia), contrast sensitivity decreases, and conditions such as cataracts, glaucoma, or dry eyes can blur or distort visual input (Owsley, 2011; Lee et al., 2013). At the cortical level, age-related slowing in interhemispheric signal transmission and in the integration of visual and motor information further interferes with the precise timing between visual perception and motor execution, resulting in handwriting that is less fluid and controlled than in younger adults. Deficits in visuomotor coordination can also impair the ability to accurately judge position, size, and spatial relationships when forming letters, leading to inconsistently sized or poorly aligned text (Lu et al., 2024).

Such changes in sensorimotor integration support the explanation of the fact that handwriting is a notable example of an activity that involves the intricate cooperation of sensory input and motor output. Sensory inputs are also less dependable and take more time than motor outputs, which leads to illogical and less continuous text formation (Ho et al., 2022). It is important to shed light on these changes and how they affect the forgetting of handwriting skills and, essentially, the independence and quality of life of older adults.

### 3.3 Age-related cognitive impairments

Aging is also the cause of cognitive deficits, which contribute to the decrease in the quality of handwriting among older adults. One of the major dysfunctions in older people is that executive functions, the functions that enable planning, organizing, and initiating actions, are usually lower in older people (Idowu and Szameitat, 2023). This reduction can limit the possibilities of anticipating and coordinating the numerous delicate muscle motions involved in the writing process, thus resulting in chaotic and fluctuating handwriting (Rosenblum, 2013). It becomes challenging for older people to provide the mental coordination of writing text, including making different letters, particularly when using the handwriting approach to produce quality texts in large quantities or even in sophisticated documents.

Short-term and long-term memory, which permit one to hold information for short and long periods of time respectively, both decline as a function of age as well (Palladino and De Beni, 1999; Čepukaitytė et al., 2023). Precise handwriting, in turn, depends on the retention of graphic forms by the human hand, the spatial and temporal organization of written language, and the observation of grammar rules (Cerni and Job, 2022, 2024). As the memory capacity reduces with age, it becomes increasingly difficult for people to manage these details, this may lead to mistakes and instances of omission and, in general, reduced quality of the texts produced in writing tasks (Bourke and Adams, 2003).

Furthermore, research suggests that people have a finite amount of attention to allocate to specific tasks, and this capacity is often more limited in older adults (Commodari and Guarnera, 2008). Maintaining the concentration is required for neat writing, as it ensures every letter and word written are properly shaped. Aging brings a decline in the ability to sustain attention, and as a result, older adults may find it difficult to focus when writing for an extended period (Silveri et al., 2007). This means that the quality of handwriting will fluctuate, whereby the more the writer is preoccupied, the less attention is paid to handwriting, and the texts produced are less neat and difficult to follow when the writer is tired or distracted.

These cognitive deficits further underscore the importance of preventing handwriting decline in aging by considering both motor and cognitive aspects (Asci et al., 2022). Among the latter, processing speed plays a particularly critical role: age-related slowing in the rate at which information is perceived, integrated, and transformed into motor output can significantly reduce handwriting fluency, timing, and coordination (Salthouse, 1996; Demaree et al., 1999). Similar effects are observed in neurodegenerative conditions such as MS, where reduced information-processing efficiency contributes to poorer handwriting control and increased latency in motor execution (Benedict et al., 2020). For this reason, interventions designed to improve handwriting in older adults should also target not only physical and sensory abilities but also cognitive functions such as processing speed, attention and executive control, offering a comprehensive approach to maintaining writing proficiency (Table 1).

**Table 1:** Age-related handwriting changes and associated kinematic, sensory, and cognitive factors.

Domain	Age-related alteration	Kinematic features	Underlying mechanisms	Clinical relevance
Motor control	Reduced dexterity and movement smoothness	Slower writing velocity, increased variability, reduced fluency	Age-related decline in motor cortex efficiency and basal ganglia modulation	Reduced legibility and writing efficiency
Sensorimotor integration	Impaired sensory feedback	Increased spatial errors, inconsistent trajectories	Reduced proprioceptive and tactile sensitivity	Difficulty in maintaining accuracy
Muscular function	Sarcopenia and fatigue	Reduced pressure control, unstable grip	Muscle weakness and reduced endurance	Writing fatigue and reduced independence
Visual–motor coordination	Decline in visuomotor integration	Poor alignment, variable letter size	Age-related visual and visuospatial decline	Reduced readability
Cognitive processing	Slower processing and executive control	Increased pauses, reduced rhythm	Decline in attention and executive function	Slower and less efficient writing

The table summarizes the main handwriting alterations reported across studies, highlighting characteristic kinematic, sensory, and cognitive features associated with aging. Representative references supporting these findings are discussed in the corresponding sections of the manuscript.

## 4 Handwriting alterations in neurodegenerative diseases

Given the complexity and multidimensional nature of handwriting alterations, objective and scalable assessment methods are increasingly needed (Impedovo and Pirlo, 2019; Vessio, 2019). The neural and behavioral changes associated with aging and neurodegenerative conditions can increasingly be explored through digital handwriting analysis. Recent advances in tablet-based kinematic acquisition, signal processing, and machine learning have facilitated the transition from predominantly qualitative observation toward more quantitative and objective assessment of motor and cognitive function. These approaches allow the characterization of both shared and condition-specific alterations in handwriting, reflecting common and distinct disruptions in distributed neural networks (Planton et al., 2013; Karimpoor et al., 2018). Although specific disorders present characteristic handwriting patterns, several alterations are also observed across conditions. To improve diagnostic clarity and facilitate comparison across studies, the main disease-related handwriting features are summarized in Table 2.

**Table 2:** Disease-specific handwriting characteristics across major neurodegenerative conditions.

Condition	Core handwriting alterations	Kinematic markers	Neural and cognitive correlates	Clinical relevance
Parkinson’s disease	Micrographia, tremor, reduced fluency	Reduced amplitude, increased stroke duration, tremor oscillations	Basal ganglia dysfunction and impaired motor timing	Early detection and disease monitoring
Alzheimer’s disease	Spatial disorganization, irregular letter formation	Increased variability, pausing, irregular spacing	Visuospatial and executive dysfunction	Cognitive screening and progression tracking
Multiple sclerosis	Fragmented and inconsistent writing	Variable velocity, pressure instability	Demyelination and impaired sensorimotor integration	Monitoring motor decline and rehabilitation

The table summarizes the main handwriting alterations reported across studies, highlighting characteristic kinematic and functional features associated with each condition. Representative references supporting these findings are discussed in the corresponding sections of the manuscript.

### 4.1 Parkinson’s disease

Handwriting analysis may provide useful complementary information in the evaluation of early motor changes associated with PD, a neurodegenerative disorder characterized by slowness of movement and tremors (Impedovo and Pirlo, 2019). Another documented symptom of PD in handwriting results from the shrinking size and restricted pen movements when writing text, a condition known as micrographia (Shukla et al., 2012). This problem reflects limited coordination and variability in fine motor control, particularly in the fingers and hands, arising from the degeneration of dopaminergic neurons in the basal ganglia (Eklund et al., 2022). As the illness advances, micrographia increases, making it an indicator of PD.

Another motor-related sign is the tremor observable in the handwriting of PD. These tremors may cause rhythmic tapping movements of the pen or pencil, producing uneven, wavy lines and variable stroke thickness (Mascia et al., 2022). They are most evident at the initiation of writing and can affect the overall legibility of the text. In addition, disturbances in letter shape and size, such as irregular or fluctuating forms, may occur in individuals with primary progressive aphasia associated with PD, reflecting higher-level planning deficits (Letanneux et al., 2014).

Although handwriting alterations in PD primarily stem from basal ganglia dysfunction, studies also indicate involvement of cerebellar circuits in motor timing and feedback regulation. Functional imaging suggests that the cerebellum may become hyperactive in PD, potentially as a compensatory mechanism for basal ganglia impairment (Wu and Hallett, 2013). Moreover, cerebellar lesion studies report handwriting abnormalities such as irregular spacing, poor temporal coordination, and tremulous strokes (Bastian et al., 1996; Manto, 2008), underscoring the interconnected roles of the basal ganglia and cerebellum in fine motor control and feedback processing.

Thus, handwriting analysis in PD is not only useful for evaluating the nature and severity of the disorder and its applicability for differential diagnosis, but also for monitoring disease progression and treatment efficacy. The assessment of micrographia, dysgraphia, or writing tremors may help evaluate the effectiveness of medication adjustments or other therapeutic interventions (Bidet-Ildei et al., 2011; Nackaerts et al., 2017; Müller and Harati, 2020). Overall, handwriting analysis may represent a safe and noninvasive complementary tool for detecting early motor changes in PD.

### 4.2 Alzheimer’s disease

Altered calligraphy can be observed in AD as an example of topographical disorientation or spatial disorientation characterized by progressively developing impairments in memory and thinking (Silveri et al., 2007). A distinct and glaring aspect of handwriting in AD patients is what is referred to as spatial disorientation; this is where the text material is irregularly spaced or aligned on the page inappropriately (Fernandes et al., 2023; Cilia et al., 2024). This disorientation is suggestive of a dyslexic pattern characterized by defective visuospatial ability, the ability to orient with the shapes formed and position letters and words within them.

Another is the formation of letters; most often, letters are poorly shaped, which is also typical for patients with AD. It may be called an irregular outline or ill-defined as it does not share the sharp, clean margins an adult with no dyslexia exhibits (Neils-Strunjas et al., 2006). The distortions mentioned here concerning the actual size and shape of the letters may stem from declining fine motor skills and dexterity, as well as from problems with vision and the processing of graphic information.

Para-aphasia, which refers to an impairment in language expression, is also manifested through unsteady stroke pressure, which is a feature of AD patients; the pressure that is applied to the writing implement varies and is not regulated. The necessity of constantly coarse-tuning the position of the stylus over the paper contributes to the irregularity, and this may cause variations in the pressure being applied, particularly in normal writing (Fernandes et al., 2023). This could make the text appear faint or unevenly shaded. These changes in stroke pressure are correlated with the other characteristics of AD, such as deterioration in executive function and motor coordination.

Handwriting analysis is beneficial in uncovering the cognitive neuromotor alterations observed in AD, including the progression and severity of the illness. These handedness characteristics are useful in observing which early behaviors predict potential cognitive decline and tracking changes longitudinally (Forbes-McKay et al., 2014).

### 4.3 Multiple sclerosis

MS is a chronic neurological condition that is characterized by the degeneration of the myelin sheath that surrounds nerve fibers, impairing communication between the brain and body (Younger, 2023). One of the often-overlooked manifestations of MS is its effect on handwriting, as a common feature in MS is an impaired hand function caused by deterioration of hand dexterity (Lamers et al., 2014), thus limiting the possibility of fine control of finger movements. As MS progresses, motor symptoms such as weakness, spasticity, tremors, and reduced speed can significantly impact handwriting. A common feature in the handwriting of MS patients is indeed irregularity in stroke production, fragmented velocity, unsteady pen pressure, reduced writing speed, and inconsistent letter formation (Bisio et al., 2017), as shown also by digital writing aids (Lam et al., 2021).

Handwriting analysis can be a useful tool in diagnosing and monitoring MS, particularly in the early stages of the disease when symptoms may be subtle. Variations in handwriting, such as the onset of tremors, difficulties in forming consistent letters, or irregular spacing between words, can help clinicians identify motor and cognitive impairments linked to MS and tailor treatment plans accordingly. Additionally, longitudinal handwriting assessments can be used to track the progression of the disease, providing insight into how cognitive and motor functions evolve in the same patient.

## 5 Digital tools for assessing and improving handwriting

Recent technological advances have progressively shifted handwriting research from descriptive observation into a quantitative, data-driven field. Digital tablets, styluses, and remote monitoring tools now allow high-resolution recording of movement dynamics, while machine-learning algorithms may help identify disease-specific patterns with increasing precision (Tolosana et al., 2017; Impedovo and Pirlo, 2019). In addition to assessment, adaptive digital tools, such as ergonomically designed pens, guided templates, and smart styluses that provide real-time feedback, may offer new possibilities for maintaining handwriting ability in older adults and clinical populations.

### 5.1 Tablets and styluses

**Figure 2:**
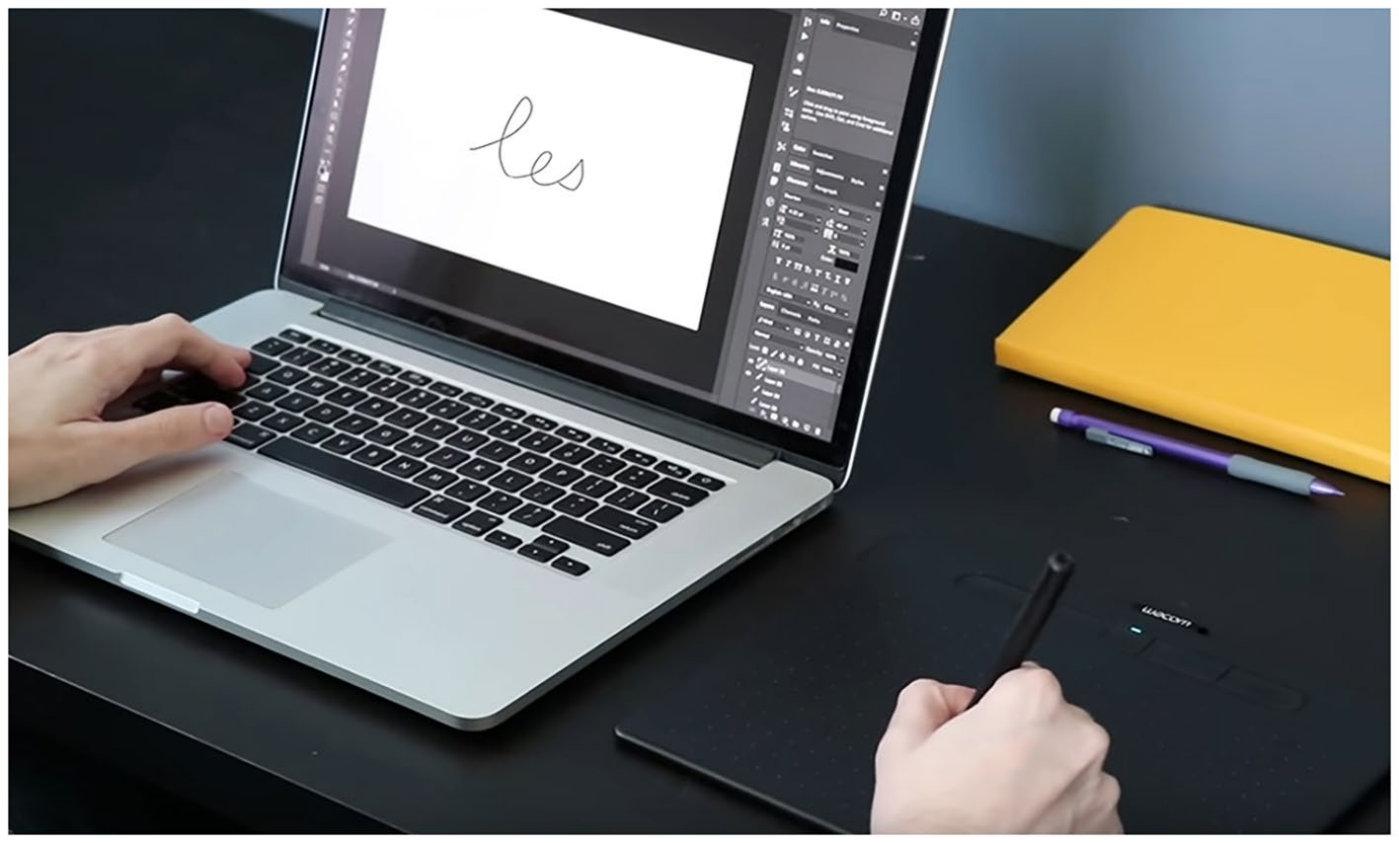
Handwriting data acquisition using a tablet-based system. As an example, the word “les” is written with a stylus on the tablet (on the right side) and graphically displayed on a laptop (on the left side).

**Figure 3:**
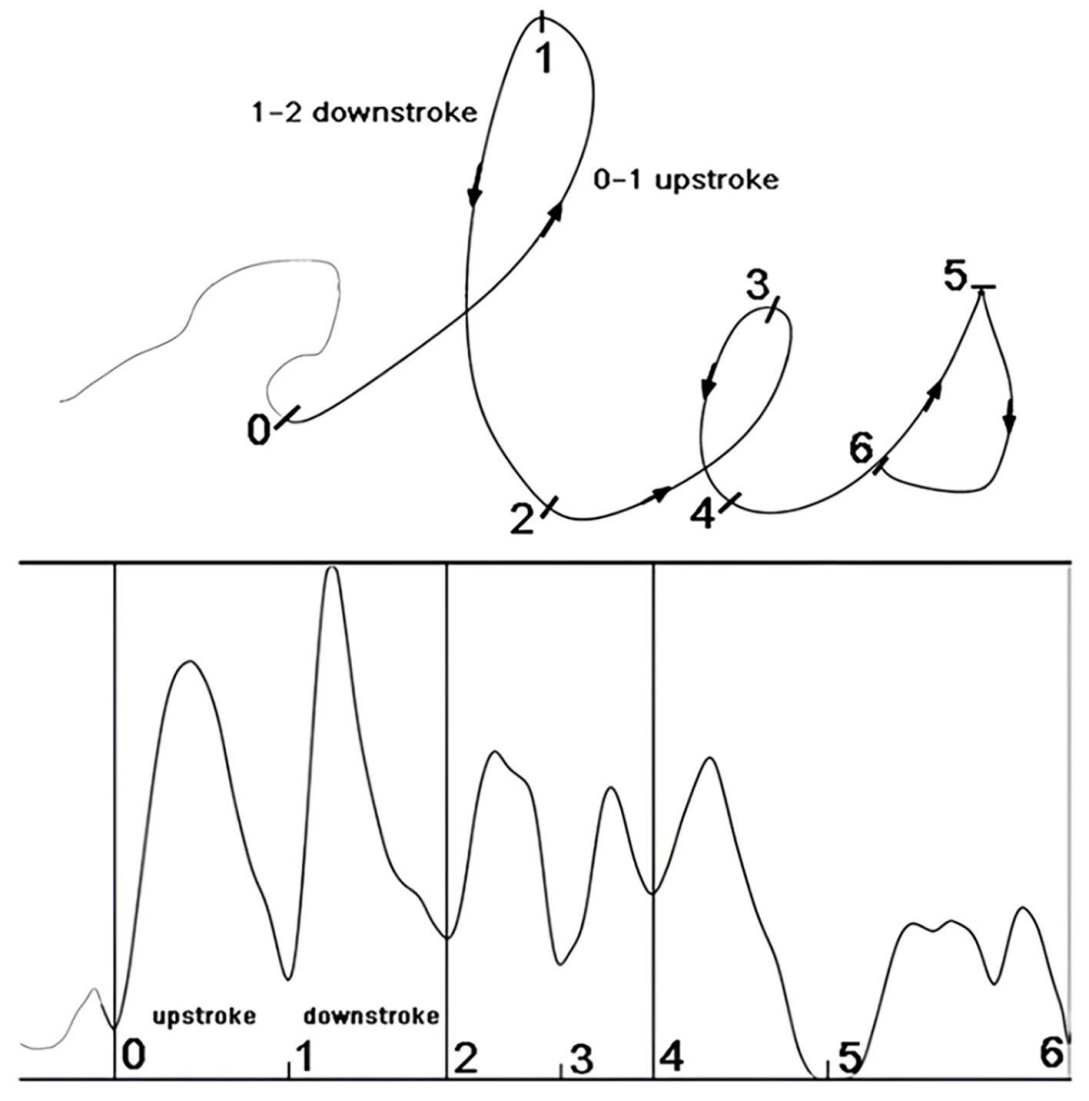
Automated segmentation of upstrokes and downstrokes. The trajectory and velocity profile of the word “les” are illustrated as an example. The figure is reproduced from Guinet and Kandel (2010), with permission.

Digital technology has greatly shifted the approach to handwriting assessments in a way that is much more accurate and detailed. In such a revolution, tablets and styluses are usually right on the front lines (Figure 2), equipped with precise tools for comprehensively capturing handwriting metrics (Prattichizzo et al., 2015; Tolosana et al., 2017). These devices can record features, such as speed, frequency, pressure, and the dynamics of different strokes with high accuracy (Figure 3). However, since the velocity and acceleration of pen movements can also be recorded, one can identify the dynamics in the formation of handwriting by precisely measuring the fluidity and rhythm of the handwriting (Guinet and Kandel, 2010; Chesnet et al., 2024; Vysakha et al., 2024). Interestingly, the amount of curvature and length of a particular stroke can show different dynamics of motor planning.

These detailed metrics are especially useful in evaluating the degree of finger dexterity loss with age, as the analysis based on such metrics reveals specific regions of handwriting degradation. For instance, variations in stroke pressure and speed are early signs of neurodegenerative diseases like PD (Heremans et al., 2016). In this regard, the details accrued by tablets and styluses suppose better sensitivity to handwriting processes, which, in turn, can enhance early detection of handwriting problems and individualized interventional approaches (Nackaerts et al., 2017).

In addition to these kinematic measures, recent research has increasingly focused on kinetic variables that provide complementary information on motor control. Kinetic variables, such as pen pressure and grip force, represent important but often underexplored aspects of handwriting performance (Rosenblum et al., 2003; Drotár et al., 2016). Digital tablets and styluses now enable the continuous recording of pressure profiles during writing, allowing the quantification of force modulation, variability, and stability (Vessio, 2019). These measures reflect neuromuscular control, sensorimotor integration, and the ability to adapt motor output to task demands (Wolpert et al., 2011).

Alterations in handwriting pressure have been reported in both healthy aging and neurodegenerative conditions (Rosenblum et al., 2003; Vessio, 2019). Older adults may show increased variability and reduced adaptability of pressure, likely related to declines in proprioception, muscle strength, and sensory feedback (Rosenblum et al., 2013). Similarly, individuals with Parkinson’s disease, Alzheimer’s disease, and Multiple Sclerosis may exhibit abnormal pressure regulation, reflecting impairments in basal ganglia function, executive control, and sensorimotor integration (Letanneux et al., 2014; Drotár et al., 2016; Bisio et al., 2017).

### 5.2 Remote monitoring

Digital technology has also facilitated the monitoring of handwriting, offering valuable tools for both healthcare professionals and patients. Font, style, and movement dynamics can now be assessed outside the clinic using tablets and styluses, which are generally more familiar and user-friendly for most individuals (Asci et al., 2022; Collins et al., 2024). One advantage of this remote capacity is its potential to benefit older adults and people with limited mobility or those living in areas with restricted access to health facilities. However, it is important to recognize that some older adults may face challenges related to digital literacy, which can limit the usability of such tools. In these cases, caregiver involvement or guided digital support can play a crucial role in ensuring that remote handwriting monitoring remains accessible and effective.

Regular monitoring can enable the detection of even subtle signs of decline, providing an early indication of the onset of neurological conditions and allowing timely adjustment of management strategies. Moreover, handwriting tasks can be performed by patients in the comfort of their own homes, reducing the stress and variability that come with clinic assessment (Gopal et al., 2022). Continuous daily data collection thus provides a more comprehensive picture of a patient’s motor function, thereby making the assessment and treatment plans more accurate.

### 5.3 Data analytics

Another important development in digital handwriting assessment has been the growing use of data analytics, including machine learning approaches, to support the analysis of complex handwriting features (Impedovo and Pirlo, 2019; Asselborn et al., 2020). These methods may help to detect subtle patterns and variability in writing behavior that are not easily captured through visual inspection alone (Asci et al., 2022). However, current evidence remains limited, and further validation in large and diverse datasets is needed to determine their robustness and clinical utility. Machine learning models have been trained on handwriting datasets to detect specific features related to different neurological conditions and, as such, offer a unique method of identifying impairments at an early stage (Asci et al., 2022; Babushkin et al., 2023).

For example, specialists might determine whether changes in handwriting in patients with a natural aging process are typical or may indicate neurological disorders by using machine learning models. Preliminary studies suggest that these approaches may support the differentiation of PD, AD, or MS based on multidimensional handwriting features, particularly with respect to stroke length, curvature, pressure applied, and variations in speed (Cilia et al., 2024; Islam et al., 2024), although clinical validation remains limited. The application of data analytics may thus allow handwriting evaluation to complement traditional clinical examinations.

In addition, a sophisticated analysis of outcomes allows conditioning an individualized approach to rehabilitation due to the definition of patterns of deterioration, stability, or improvement in each case and their response to therapy. Such systems get updated with new data for the model to be retrained and made more accurate. This dynamic approach to handwriting assessment may support earlier identification of relevant changes and more personalized monitoring (Danna and Velay, 2015; Babushkin et al., 2023).

The technological innovations described above have set the stage for the clinical translation of handwriting analysis. By combining high-resolution kinematic data with machine learning and computational modeling, these digital tools are increasingly being applied to identify disease-specific motor and cognitive signatures. The following section summarizes how such approaches are being used to support differential diagnosis across major neurodegenerative conditions.

### 5.4 Adaptive writing tools

Movable writing peripherals can help healthy older adults and patients overcome the loss of precise control and reduce inefficiencies in sensory processing, allowing them to continue writing independently and with greater ease. Ergonomic pens, for instance, feature larger cushioned grips that make handling easier and writing more comfortable by reducing the strain on hand muscles and joints (Goonetilleke et al., 2009). These made-to-measure pens offer a better grip, improving pen stroke management and positively influencing writing style and document neatness.

Writing guides are also essential for maintaining standards in letter size and word spacing. They are particularly useful for those who experience disorientation or coordination problems, especially with vision (Letanneux et al., 2014; Fernandes et al., 2023). By providing an organized framework, writing guides help older adults compose more coherent and readable documents. Furthermore, digital writing aids, such as adaptive tablet styluses, can record handwriting metrics and enhance visualization in real time (Korres et al., 2021). These tools provide immediate feedback on parameters such as stroke pressure, velocity, and rhythm, allowing users and clinicians to track performance continuously. This real-time measurement capability is particularly valuable for guiding personalized interventions and adjusting training strategies as handwriting improves or declines. In this way, digital aids not only support the writing process but also function as biofeedback systems that promote motor learning and rehabilitation.

Overall, incorporating goal-directed movement and flexible writing instruments into daily practice may enable older adults to maintain basic handwriting abilities, preserve independence, and remain engaged in written communication. These strategies not only benefit functional capacities but are also critical for the overall health and well-being of the cognitive and physical systems of aging individuals (Bar-Tur, 2021).

## 6 General discussion and future directions

The preceding sections have outlined the neural, cognitive, and technological foundations of handwriting and their alterations in aging and neurodegenerative disease. This final section integrates these insights to provide a comprehensive perspective on the mechanisms, assessment methods, and clinical applications of handwriting research. By synthesizing key findings, evaluating current methodological challenges, and examining diagnostic and translational implications, this discussion highlights both the progress achieved and the gaps that remain. It also outlines future directions aimed at standardizing digital handwriting assessments, enhancing diagnostic specificity, and promoting interdisciplinary collaboration toward the use of handwriting as a reliable biomarker of neurological health.

### 6.1 Synthesis of main findings

Handwriting is a complex behavior that emerges from the dynamic interaction of motor, sensory, visuospatial, and cognitive processes within distributed brain networks. Rather than depending on isolated neural regions, writing relies on coordinated activity across cortical and subcortical systems, including the primary motor and premotor cortices, supplementary motor area, parietal regions, basal ganglia, and cerebellum (Tanji, 1994; Scott, 2003; Chouinard and Paus, 2006; Horovitz et al., 2013; Wu and Hallett, 2013). These systems interact through functional connectivity and feedback mechanisms to support motor planning, execution, error correction, and adaptation to changing task demands.

Across the adult lifespan, structural and functional changes within these networks, as well as deterioration in the white matter tracts connecting them, contribute to progressive alterations in handwriting performance (Raz et al., 2005; Seidler et al., 2010; Zapparoli et al., 2022; Iskusnykh et al., 2024). Aging is associated with declines in sensorimotor integration, proprioceptive and tactile sensitivity, visuomotor coordination, and executive functioning, leading to slower and more variable movements, reduced fluency, and increased spatial inconsistency. Evidence reviewed here indicates that these neural and behavioral alterations manifest in measurable changes in handwriting kinematics and spatial organization (Bisio et al., 2017; Asci et al., 2022; Fernandes et al., 2023).

In neurodegenerative diseases, these processes are further amplified and combined with disorder-specific mechanisms. PD, AD, and MS each affect different neural subsystems but converge in disrupting the integration of motor, sensory, and cognitive processes required for writing. As a result, handwriting alterations share common features across conditions while also presenting distinctive patterns related to the underlying pathology (Seeley et al., 2009).

Taken together, the literature reviewed in this article supports the conceptualization of handwriting as a multidimensional behavioral marker of neural function. Because writing integrates multiple domains within ecologically relevant tasks, it offers a unique opportunity to capture subtle changes associated with aging and neurodegeneration. This perspective provides a foundation for the methodological, diagnostic, and translational considerations discussed in the following sections. Recent advances in digital handwriting analysis now allow high-resolution, quantitative assessment of these changes, offering new opportunities for early detection, continuous monitoring, and rehabilitation of age- and disease-related decline (Tolosana et al., 2017; Gopal et al., 2022; Babushkin et al., 2023; Islam et al., 2024).

### 6.2 Critical reflections on current methodologies

Despite several advances, technical and methodological challenges continue to limit the widespread clinical adoption of digital handwriting tools. One major issue is hardware variability: differences in sampling rate, spatial resolution, latency, and pressure sensitivity across tablets and styluses can affect the precision of extracted parameters and hinder cross-study comparisons (Korres et al., 2021). Additionally, the absence of standardized experimental protocols, from task design and calibration to feature extraction and data interpretation, introduces inconsistencies that reduce reproducibility and generalizability across laboratories and clinical settings (Guinet and Kandel, 2010; Chesnet et al., 2024).

Besides technological aspects, user-related and clinical factors also deserve attention. Handwriting performance is influenced not only by neurological and cognitive processes but also by peripheral and sensory conditions that may act as confounders in both research and clinical contexts (Rosenblum et al., 2003, 2013). Musculoskeletal disorders such as arthritis, tremor of non-neurological origin, and age-related joint stiffness can significantly affect grip stability, pressure control, and fine motor execution, potentially mimicking or masking disease-related alterations (Carmeli et al., 2003). Visual impairments, including reduced acuity, contrast sensitivity, and ocular pathology, may further influence spatial organization and letter formation (Whittaker and Lovie-Kitchin, 1993).

Sociocultural and educational variability may also contribute to differences in handwriting characteristics. Literacy level, writing habits, and cultural conventions can influence motor patterns and spatial organization, particularly in heterogeneous populations (Castro-Caldas et al., 1998). Moreover, some older adults may have limited familiarity with digital interfaces, and digital literacy may affect both task performance and usability. Providing caregiver support, guided interfaces, and adaptive task designs may help reduce these barriers and improve accessibility (Bar-Tur, 2021; Gopal et al., 2022).

Another important consideration concerns scalability and real-world implementation. Many current studies rely on high-fidelity digitizing tablets and specialized acquisition systems that enable precise kinematic and kinetic measurements but may be difficult to deploy in routine clinical practice (Drotár et al., 2016). In contrast, consumer-grade devices offer greater accessibility and scalability but differ in technical specifications, which may affect measurement accuracy and comparability across settings. Practical factors such as cost, usability in older and clinical populations, integration within existing healthcare infrastructures, and compatibility with telemedicine platforms must therefore be addressed. Ensuring interoperability between devices, standardization of data formats, and secure data management will be essential to support large-scale adoption (Rieke et al., 2020).

Addressing these challenges will require consensus-based frameworks, standardized experimental protocols, and normative datasets stratified by demographic, clinical, and technological variables. The development of open benchmarking datasets and coordinated validation studies across research-grade and consumer technologies will be critical to harmonize data collection and ensure the reliability, specificity, and generalizability of digital handwriting metrics (Goldsack et al., 2020). Such efforts will be essential for translating digital handwriting analysis from controlled research environments to scalable and equitable clinical applications (Tolosana et al., 2017; Lam et al., 2021).

### 6.3 Summary of diagnostic applications across conditions

Digital handwriting analysis has revealed both condition-specific and transdiagnostic features associated with motor and cognitive dysfunction. In Parkinson’s disease (PD), handwriting is commonly characterized by micrographia, reduced pen pressure, prolonged stroke duration, and tremor-related irregularities, reflecting bradykinesia, impaired motor timing, and basal ganglia dysfunction (Letanneux et al., 2014; Thomas et al., 2017; Eklund et al., 2022; Mascia et al., 2022; Collins et al., 2024). In Alzheimer’s disease (AD), spatial disorganization, stroke discontinuity, irregular spacing, and increased pausing are frequently observed, consistent with impairments in executive and visuospatial control (Neils-Strunjas et al., 2006; Silveri et al., 2007; Fernandes et al., 2023; Cilia et al., 2024). Multiple sclerosis (MS), in contrast, often presents with irregular velocity profiles, pressure variability, reduced smoothness, and fatigue-related fluctuations, reflecting demyelination and impaired sensorimotor integration (Lamers et al., 2014; Bisio et al., 2017; Benedict et al., 2020; Younger, 2023).

Despite these characteristic patterns, substantial phenotypic overlap exists across neurodegenerative conditions, reflecting shared disruptions in distributed neural networks involved in motor control, visuospatial processing, and executive function (Wolpert et al., 2011; Planton et al., 2013). As a result, individual handwriting variables, such as writing velocity, stroke amplitude, vertical pressure, spatial variability, or in-air trajectory time, may not be sufficient to differentiate between disorders when considered in isolation (Thebaud et al., 2025). For example, reduced writing speed and increased variability can be observed in both PD and AD, whereas pressure instability and fragmented movement profiles may also occur in MS and in advanced stages of other neurodegenerative diseases.

When considering the diagnostic value of handwriting assessment, it is therefore essential to address not only sensitivity but also specificity. Differential diagnosis requires identifying multidimensional patterns rather than relying on single features (Rios-Urrego et al., 2019). For instance, micrographia and tremulous strokes remain particularly indicative of PD (Drotár et al., 2016), whereas spatial disorganization, irregular letter forms, and planning deficits are more typical of AD (Schröter et al., 2003). In MS, handwriting often exhibits variable pen pressure, reduced smoothness, and inconsistent motor output. Combining multiple kinematic, kinetic, and temporal features may improve diagnostic differentiation across disorders and provide a more robust characterization of disease-specific motor and cognitive profiles.

In addition, emerging methodological approaches are expanding the dimensionality of handwriting analysis beyond traditional two-dimensional kinematic measures. Multimodal frameworks integrating spatial, temporal, kinetic, and physiological signals, including three-dimensional movement dynamics, pressure modulation, grip force, and electromyographic (EMG) activity, offer a more comprehensive description of neuromuscular control during writing (Wang et al., 2025). These approaches may capture subtle alterations in motor coordination, fatigue, and sensorimotor integration that are not detectable using conventional metrics (Wolpert et al., 2011). As such, multidimensional handwriting assessment represents a promising direction to enhance diagnostic sensitivity, improve specificity, and support personalized monitoring across disease stages (Drotár et al., 2016). However, further validation in large and diverse populations, along with standardized acquisition protocols, will be necessary to translate these methods into routine clinical practice.

### 6.4 Neural decoding and brain-computer interfaces

Recent advances in neural recording and decoding techniques have opened new perspectives for investigating the neural mechanisms underlying handwriting. Both invasive and non-invasive approaches have demonstrated that handwriting movements and even the intended identity of characters can be decoded directly from neural activity (Willett et al., 2021). Studies using intracortical recordings have shown that complex writing trajectories and linguistic content can be reconstructed with high accuracy, highlighting the distributed representation of handwriting across motor, premotor, and language-related brain regions (Xu et al., 2025). Similarly, non-invasive techniques such as electroencephalography (EEG) have provided evidence that cortical activity patterns contain information related to handwriting dynamics and character identity (Pei et al., 2021).

These findings have important implications for both basic neuroscience and clinical translation. From a mechanistic perspective, neural decoding studies provide novel insights into how distributed brain networks encode motor planning, sensorimotor integration, and error correction during writing (Wolpert et al., 2011). From a translational perspective, these approaches support the development of brain–computer interfaces (BCIs) that may enable communication and functional restoration in individuals with severe motor impairments (Pandarinath et al., 2017; Willett et al., 2021).

In the future, neural decoding technologies may also contribute to the characterization of age- and disease-related changes in the neural control of handwriting. Such approaches could provide complementary biomarkers to behavioral and kinematic measures, potentially supporting earlier diagnosis and more personalized neurorehabilitation strategies (Rosenblum et al., 2003). However, further research is needed to validate these methods in clinical populations and to address challenges related to scalability, usability, and ethical considerations (Clausen, 2011; Nijboer et al., 2013).

### 6.5 Clinical and research implications

From a clinical perspective, handwriting analysis may complement traditional neuropsychological and motor assessments by providing objective and quantitative information on fine motor and cognitive performance (Ericsson et al., 1996; Rosenblum, 2013). Digital platforms allow repeated and ecologically valid measurements over time, which may support longitudinal monitoring and early identification of subtle functional changes. The integration of digital handwriting tools within telehealth environments could further enhance accessibility, particularly for older adults and individuals with limited mobility (Gopal et al., 2022; Islam et al., 2024). However, careful interpretation remains necessary, as handwriting performance is influenced by multiple neurological, sensory, and contextual factors.

Beyond behavioral measures, emerging neurophysiological and neuroimaging approaches may provide complementary insights into the neural processes underlying handwriting (Kandel and Spinelli, 2010; Planton et al., 2013). The integration of digital handwriting metrics with techniques such as electroencephalography or functional neuroimaging could contribute to a more comprehensive characterization of motor planning, sensorimotor integration, and cognitive control (Planton et al., 2017). Multimodal frameworks combining neural and behavioral data may improve the sensitivity and interpretability of handwriting-based biomarkers and help to capture individual variability in disease progression and compensatory mechanisms (Coravos et al., 2019; Goldsack et al., 2020).

Rehabilitation programs may also benefit from incorporating digital handwriting tasks, particularly as a means to support functional abilities, motor coordination, and cognitive engagement. Real-time feedback and adaptive task design may facilitate motor learning and the development of compensatory strategies, potentially improving daily functioning and quality of life (Kleim and Jones, 2008; Dayan and Cohen, 2011; Nackaerts et al., 2017). Nevertheless, current evidence regarding the impact of handwriting-based interventions on the progression of neurodegenerative diseases remains limited. Most studies involve small samples and heterogeneous protocols, and further randomized controlled trials are needed to clarify their long-term effectiveness. At present, such interventions should be considered supportive rather than disease-modifying. Combining handwriting-based training with cognitive interventions, assistive technologies, or non-invasive brain stimulation may represent promising directions but requires further validation (Polanía et al., 2011; Mao et al., 2021).

At the research level, integrating handwriting metrics with other digital and clinical markers, such as gait analysis, speech features, neuroimaging, and physiological measures, may contribute to the development of multidimensional phenotyping strategies in aging and neurodegenerative diseases (Gopal et al., 2022; Fernandes et al., 2023). These approaches may facilitate the identification of disease subtypes, improve patient stratification, and support more personalized monitoring and intervention.

Finally, as digital handwriting analysis systems based on artificial intelligence move toward clinical translation, ethical and societal considerations should remain central. Ensuring data privacy, informed consent, accessibility, algorithmic transparency, and equitable access to technology will be essential to maintain trust, minimize bias, and promote responsible innovation (Ho et al., 2022).

## 7 Future directions and conclusions

The next phase of handwriting research will depend on standardization and scalability. Establishing unified protocols, open normative databases, and interoperable data formats will facilitate cross-cohort validation and meta-analytic synthesis (Tolosana et al., 2017; Lam et al., 2021). Incorporating artificial intelligence and machine learning approaches may enable automatic detection of disease-specific signatures and personalized tracking of progression (Babushkin et al., 2023; Cilia et al., 2024; Islam et al., 2024).

Longitudinal studies are essential to confirm the predictive value of handwriting biomarkers for conversion from mild to severe stages of neurodegenerative disease (Fernandes et al., 2023; Collins et al., 2024). Finally, progress will require interdisciplinary collaboration between neuroscientists, clinicians, engineers, and rehabilitation specialists to translate digital handwriting analysis from research to real-world healthcare (Lee et al., 2022).

In conclusion, handwriting represents a sensitive and integrative window into the aging brain. Understanding the neural mechanisms underlying handwriting decline may contribute to earlier identification of neurodegenerative processes and support the development of targeted and supportive technology-assisted interventions. The integration of digital innovation, standardized methodologies, and multimodal behavioral and neural markers may contribute to the development of robust and scalable biomarkers for early diagnosis, longitudinal monitoring, and personalized neurorehabilitation across aging and neurodegenerative conditions, primarily aimed at improving functional abilities and quality of life.
